# Proteomic Analysis of Uterine Tissues During Peri-Implantation Period in Mice with Experimentally Induced Adenomyosis that Treated with anti-Ngf: Implications for Cell-Cell Adhesion and Metabolic Processes

**DOI:** 10.1007/s43032-020-00262-y

**Published:** 2020-07-16

**Authors:** Yan Li, Dan Zhang, Bailing Jin, Lan Xia, Aijun Zhang

**Affiliations:** Reproductive Medical Center of Ruijin Hospital, School of Medicine, Shanghai Jiao Tong University, 197 Ruijin 2nd Road, Shanghai, 200025 China

**Keywords:** Adenomyosis, NGF-neutralizing antibody, Endometrial receptivity, Mass spectrometry analysis, Integrin-related proteins

## Abstract

Nerve growth factor (NGF) has been verified to be expressed with higher level in adenomyosis uteri, and its neutralizing antibody has a strong inhibitory influence on inflammation. The present study aimed to explore the effect of anti-NGF on the expression of proteins in uteri of mice-induced adenomyosis and assessed its potential role in improving pregnancy rate. In this study, we established a mouse model of adenomyosis and administrated NGF-neutralizing antibody into mice. The mass spectrometry (MS) analysis of the uteri during the implantation window was performed to explore the essential proteins participating in therapy. Besides, embryos of healthy mice were transferred into the uteri to assess the implantation rate. The results of MS analysis demonstrated that 119 proteins were changed in the adenomyosis group compared with control group, and 126 proteins were differentially expressed in the anti-NGF group compared with the adenomyosis group (fold change > 1.5, *P* < 0.05. After performing cluster analysis using Mfuzz package, we found that a group of proteins participated in cell-cell adhesion and metabolic processes, which were attenuated in the adenomyosis group, while those were successfully recovered by anti-NGF treatment. Western blotting confirmed that the expression levels of integrin alpha-1 (ITGA1), integrin beta-1 (ITGB1), laminin subunit gamma-1 (LAMC1), and creatine kinase M-type (CKM) were decreased in adenomyosis group, whereas those levels were elevated after anti-NGF treatment. Embryo implantation rate in the adenomyosis group was significantly decreased compared with that in the control group (2.31% vs. 26.15%, *P* < 0.001) and anti-NGF treatment slightly enhanced the embryo implantation rate in mice with experimentally induced adenomyosis (9.23% vs. 2.31%, *P* = 0.017). Our results revealed that anti-NGF therapy can improve fertility of mice with experimentally induced adenomyosis, possibly through promoting integrin-related proteins.

**Electronic supplementary material:**

The online version of this article (10.1007/s43032-020-00262-y) contains supplementary material, which is available to authorized users.

## Introduction

Adenomyosis is a benign gynecological disorder frequently observed in women in their reproductive age. Epidemiological and clinical evidences demonstrated that 22% of infertile women who aged less than 40 years old and underwent assisted reproductive technology had adenomyosis [1]. Besides, a reduction in clinical pregnancy rate (40.5% for adenomyosis vs. 49.8% for controls) was found in patients with adenomyosis who underwent in vitro fertilization [2]. A noticeably reduced rate of spontaneous pregnancy was reported in women with concomitant adenomyosis who underwent surgical treatment of rectovaginal and bowel endometriosis compared with those without adenomyosis (11.9% vs. 43.0%) [3]. A number of scholars demonstrated that there were abnormal aggregation of inflammatory immune cells and cytokines in adenomyosis nodes, including dendritic cells, macrophages, neutrophils, tumor necrosis factor-α (TNF-α), interleukin 6 (IL-6), IL-8, nerve injury-induced protein1, and cyclooxygenase-2 (COX-2) [4–6]. In addition, disturbed uterine peristalsis, anatomical distortion of uterine cavity, altered uterine oxidative stress environment, and changing endometrial steroid metabolism may all contribute to the impaired endometrial receptivity in adenomyosis patients [7–9]. Generally, adenomyosis is treated with hormonal medications, as well as fertility-sparing radiological and surgical techniques [10, 11]. However, potential therapeutic targets for improving adenomyosis-related fertility were still needed and studied [12, 13].

Studies revealed that adenomyotic nodules were rich in neurogenic factors (e.g., nerve growth factor (NGF)) [14, 15]. The expression level of NGF was found to be associated with the severity of adenomyosis [16]. At the same time, the increased levels of NGF and its receptors were noted to be associated with the inflammation status and augmented innervation in the uterine adenomyosis [16]. NGF can stimulate the proliferation and increase aromatase expression of endometrial stromal cells [17]. As NGF has multiple functions on cell proliferation, immunomodulation, innervation, and hyperalgesia, an anti-NGF treatment is highly beneficial to attenuate pain/inflammatory signals [18]. Given that NGF exaggerates inflammatory responses and is highly expressed in the adenomyotic lesions, we, in the present study, attempted to indicate whether anti-NGF medication could be beneficial for pregnancy rate.

To examine this hypothesis, we established a mouse model of adenomyosis, with administration of the NGF-neutralizing antibody. We here utilized tamoxifen to establish a mouse model of adenomyosis [19]. The proposed mechanism is that the paracrine signaling inhibits differentiation of uterine myocytes in the mesenchyme, facilitating invagination of the basalis endometrium into the myometrium [19–23]. Typically, the depth and area of the adenomyosis nodes in uteri of the mice with adenomyosis may constantly increase with the elevation of age [16]. We engaged embryo transfer of healthy mice rather than natural mating because the tamoxifen, which was used to induce model, may have a negative influence on the quality of oocyte and ovulation. The mass spectrometry (MS) analysis of the uteri was herein performed, and four favorable proteins were validated by Western blotting and immunohistochemistry (IHC).

## Materials and Methods

### A Mouse Model of Adenomyosis

Neonatal Female Institute of Cancer Research (ICR) mice (SCXK 2013-0016, SLAC laboratory animal Co. Ltd., Shanghai, China, bought with their mother mice) were raised in negative pressure isolators at 20–25 °C with a 12-h light/dark cycle. All mice were from 1 mother mice and randomly grouped using a random number table. To establish a mouse model of adenomyosis, neonatal mice were orally administered with 2.7 μmol/kg tamoxifen (Shanghai Fudan Forward Co. Ltd., Shanghai, China) suspended in a peanut oil/lecithin/condensed milk mixture on days 2 to 5 after birth (day of birth was day 1, inject the mixture into the mouth of suckling mouse slowly with micropipettor). The control mice received no therapy. The neonatal mice were raised with their mother for 1 month and then separated for the following study. All the above-mentioned experimental procedures were approved by the Ethics Committee of Ruijin Hospital, Shanghai Jiaotong University School of Medicine (Shanghai, China).

### Anti-NGF Therapy

Mice with experimentally induced adenomyosis received anti-NGF treatment at the age of 27~28 weeks old (*n* = 20 per each group). Anti-NGF group was administered with 10 mg/kg of NGF-neutralizing antibody (Sino Biological Inc., Beijing, China) through intraperitoneal (i.p.) injection (injection was given every 5 days, in total 3 treatments/mouse) [24]. Equal volumes of vehicle solutions were given to adenomyosis vehicle group (0.01 ml/g i.p. normal saline). Control group received no treatment.

### Pseudopregnancy

When the mice were over 29 weeks old and within 5 days of anti-NGF therapy (or vehicle solution), donors were prepared. The pregnant mare serum gonadotropin PMSG (ProSpec, Rehovot, Israel) was intraperitoneally injected into all mice for 5–10 IU at around 4 pm on the first day; 48 h later, 5 IU of human chorionic gonadotropin (hCG, Livzon Pharmaceutical Group Inc., Zhuhai, China) was injected. Then, surrogate pseudopregnant ICR females, used as recipients of embryos, were mated with vasectomized ICR males (Nanjing Biomedical Research Institute, Nanjing, China; age, 3–4 months old), and the vaginal plug was observed on the next morning. On the next morning, the female mice were examined for vaginal plugs, and the plug-positive females were considered to be at day 0.5 of pseudopregnancy.

### Embryo Transfer

On day 3.5, embryos were collected from the superovulated C57BL/6 donor females (SLAC Laboratory Animal Co. Ltd., Shanghai, China; age, 8–10 weeks old). The embryos were surgically transferred into the three groups of mice on pseudopregnant day 2.5 (6 or 7 embryos to each uterine horn, 13 embryos for 1 mouse). Recipient mice were anesthetized with tribromoethanol through intraperitoneal (i.p.) injection(300 mg/kg) [25] before embryo transfer. On designated pseudopregnant days 13–14, mice were sacrificed to observe the implanted embryos. The percentage of the number of existing embryos to total implanted number was calculated for each group as well.

### Sacrifice

The duration of the animal experiment were 32 weeks (from D2 dose with tamoxifen to pseudopregnant day 13–14). Some of the pseudopregnant mice were sacrificed at the day of plug-positive (*n* = 5~8 per each group). Additionally, other pseudopregnant mice used for embryo transfer (*n* = 10 per each group) were sacrificed on designated pseudopregnant days 13–14. After sacrifice, the uteri of mice were collected either for molecular biological detecting or counting the implanted sites. In view of the potential severe pain caused by adenomyosis or embryo transfer surgery, we set humane endpoints for mice with one of the following conditions: (1) weight loss more than 25%, (2) continuous arch back, and (3) tremor, cramps, lying down, or screaming more than 1 h. Twelve mother mice were sacrificed when they separated with the experimental mice. All mice were sacrificed by cervical dislocation, and cardiac arrest was considered fatal.

### MS

For each group, 3 clustered samples were used for subsequent analysis. The purified protein samples from uteri were dialyzed using a Slide-A-Lyzer Dialysis Cassettes (Pierce Biotechnology Inc., Rockford, USA) with molecular weight value of 10 kDa. The dimer and trimer/tetramer solutions were further washed before MS analysis. Each sample was centrifuged at 14.000 g for 15 min at 4 °C. The centrifuged protein samples were processed with a Micromass Nano-ESI-TOF MS system (positive ion mode; Waters Corp., Milford, MA, USA). The source was operated at an elevated pressure, and the created droplet size was 1 μm. Under an injection volume of 2 μl, mass spectra were recorded with a capillary voltage of 1.2 kV and a cone voltage of 150 V. All spectra were calibrated using 25 mg/ml cesium iodide solution. Product ions were analyzed using an orthogonal TOF analyzer, and MS data were processed with the MaxQuant software (version 1.3.0.5). Proteins were identified by searching MS and MS/MS data of peptides against a decoy version of the International Protein Index (IPI) database (version 3.87; European Bioinformatics Institute, Cambridge, UK). The false discovery rate (FDR) for peptides and protein groups was set to 0.05. FDR was calculated by the number of hits from the reverse database divided by the number of forward hits. Label-free quantification was performed using intensity determination and normalization algorithm with the help of the MaxQuant system.

### Western Blot Analysis

After protein quantization, 15 μg of protein was loaded, resolved on 10% sodium dodecyl sulfate (SDS)-polyacrylamide gels, electrotransferred onto nitrocellulose membranes, and blocked with serum. The membranes were incubated overnight with rabbit antibodies against ITGA1 (1:1000 dilution; ab181434; Abcam, Cambridge, UK), rabbit antibodies against ITGB1 (1:5000 dilution; ab183666; Abcam, Cambridge, UK), rabbit antibodies against LAMC1 (1:1000 dilution; ab233389; Abcam, Cambridge, UK), and rabbit antibodies against CKM (1:1000 dilution; sc-365046; Santa Cruz Biotechnology, Inc., Dallas, TX, USA). After 3 times of washing, secondary antibodies were added. Blots were detected by an enhanced chemiluminescence (ECL) reagent (ECL-Plus/Kit; Amersham, Piscataway, NJ, USA). Besides, β-actin (1:1000 dilution; Sigma-Aldrich, St. Louis, MO, USA) was used as loading control.

### IHC

After the mice were sacrificed, uteri were resected and immediately fixed in 10% formalin, embedded into paraffin, cut into 5 um sections, and mounted on slides. The expression levels of ITGA1, ITGB1, LAMC1, and CKM were detected by IHC. The primary antibodies were used as follows: ITGA1 (1:100 dilution; Abcam, Cambridge, UK), ITGB1 (1:300 dilution; Abcam, Cambridge, UK), LAMC1 (1:300 dilution; Abcam, Cambridge, UK), and CKM (1:100 dilution; Santa Cruz Biotechnology, Inc., Dallas, TX, USA) at 4 °C overnight. On the next day, sections were incubated with biotinylated goat anti-rabbit IgG (Zhongshan Golden Bridge Biotechnology Co., Ltd., Beijing, China) for 15 min, and then with streptavidin-peroxidase complex (Zhongshan Golden Bridge Biotechnology Co., Ltd., Beijing, China) for another 15 min at room temperature. Eventually, the sections were incubated with 3,3′-diaminobenzidine (Zhongshan Golden Bridge Biotechnology Co., Ltd., Beijing, China) at room temperature for 3 min. Negative controls were analyzed on sections incubated without primary or secondary antibody. There was no mark in any groups and the control sections. The sections were visualized using a microscope (BX51; Olympus, Tokyo, Japan) and were photographed with a digital camera (DP71; Olympus, Tokyo, Japan). Quantitative analysis was carried out using five random fields at magnification of × 200 for each endometrial slice. The background light of each photo was consistent. Dark brown staining indicated a positive reaction.

### Statistical Analysis

All data were analyzed by SAS 6.12 software (SAS Institute Inc., Cary, NC, USA) and expressed as mean ± standard deviation (SD). The comparison was made by using the Chi-square test, and the continuous data were compared with one-way analysis of variance (ANOVA), and LSD (least significant different method) is used to further compare. The pregnancy rate was calculated using the number of pregnant individuals divided by all recipient animals, and the rate of embryo implantation was calculated using the number of successfully implanted embryo divided by all transferred embryos. *P* < 0.05 was considered statistically significant.

## Results

### Anti-NGF Treatment Enhanced the Embryo Implantation Rate in the Established Mouse Model of Adenomyosis

**Fig. 1:**
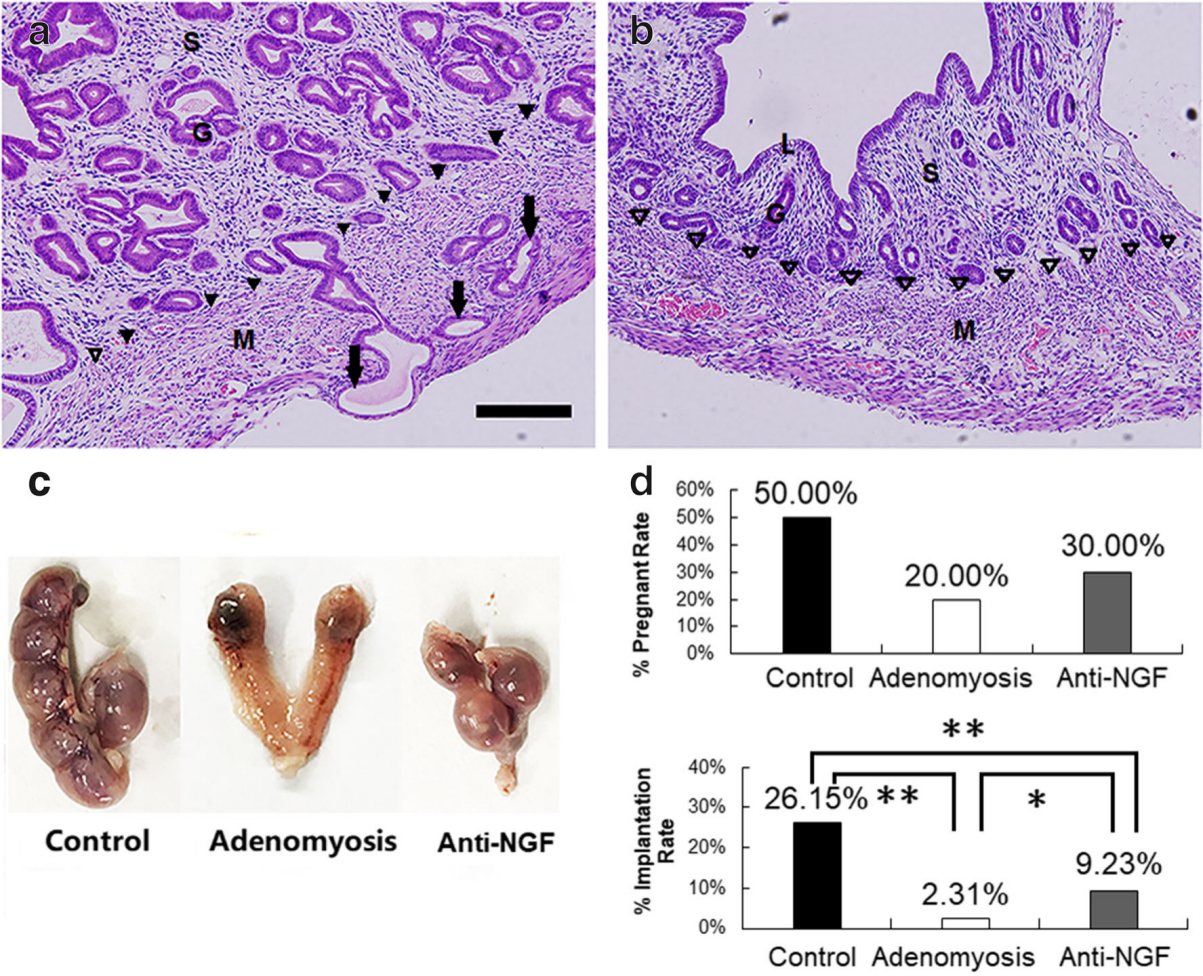
Adenomyosis mice model and embryo transfer. **a** Uterus of mice with experimentally-induced adenomyosis (M, myometrium cell; L, luminal epithelium; G, glandular epithelium; S, stromal cell; arrows, ectopic endometrium; blank triangle, complete endometrium-myometrium boundary; black triangle, impaired endometrium-myometrium boundary; Bar = 100 μm). **b** Uterus of mice as control. **c** Typical cases of implanted embryos in three groups. **d** The pregnancy rate and embryo implantation rate in control, adenomyosis, and anti-NGF groups (*n* = 10, *P < 0.05, ** *P* < 0.001)

Based on pathological examination, adenomyosis was diagnosed, and all mice, which received tamoxifen, were confirmed to develop adenomyosis. It shows impaired endometrium-myometrium boundary and the presence of ectopic endometrial glands and stroma located within the myometrium (Fig. 1a). None of the mice in Control group exhibits adenomyosis nodes in the uteri (Fig. 1b). The embryo implantation sites are dissected to observe the pregnancy and implantation rates on days 13–14 (Fig. 1c, d). The pregnancy rates were 50%, 20%, and 30% in control, adenomyosis, and anti-NGF groups, respectively(*n* = 10, *P* > 0.05). The implantation rate in the adenomyosis group significantly decreased compared with that in the control group (2.31% vs. 26.15%, Chi-square = 30.28, *P* < 0.001); besides, the embryo implantation rate of mice with adenomyosis markedly increased in the anti-NGF group compared with that in the adenomyosis group (9.23% vs. 2.31%, Chi-square = 5.73, *P* < 0.05).

### Proteins Associated with Cell-Cell Adhesion and Metabolic Processes Were Differentially Expressed in the Recovery of Adenomyosis by Anti-NGF Therapy

**Fig. 2:**
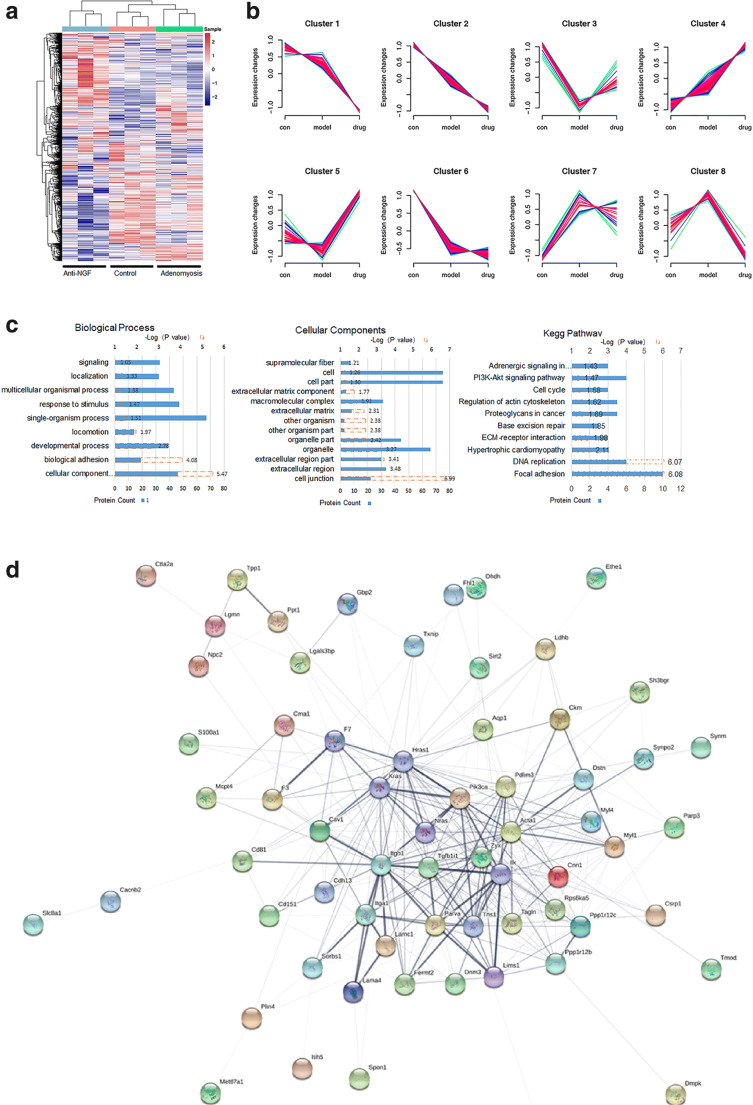
Differentially expressed proteins in different groups by MS analysis. **a** The hierarchical cluster analysis (HCA) of 4242 proteins(*n* = 3). **b** The Mfuzz package was used for HCA, in which 8 clusters were screened with the threshold of fold change > 1.5 and *P* < 0.05. **c** Results of functional enrichment analysis based on cluster 3/5 included proteins, GO terms, biological processes, cellular components, and KEGG pathway enrichment. **d** Protein-protein interaction network based on cluster 3/5 using STRING database

The tryptic peptides from endometrial protein samples of uteri were analyzed by liquid chromatography-mass spectrometry (LC-MS)/MS. Differential expression level was set to fold change > 1.5 (either upregulation or downregulation), and *P* value should be less than 0.05 in the Student’s *t* test. For each group, 3 clustered samples were used for subsequent analysis. There were 4242 peptides identified, of which 119 proteins were changed in the adenomyosis group compared with the control group, and 126 proteins were differentially expressed in the anti-NGF group compared with the adenomyosis group. Afterward, the hierarchical cluster analysis (HCA) is performed, in which the heat map is depicted in Fig. 2a. Furthermore, the Mfuzz package is used to carry out cluster analysis, and 8 typical clusters are achieved (Fig. 2b) using the threshold of fold change > 1.5 and *P* < 0.05. With further studying clusters 3 and 5, we found those decreased proteins in adenomyosis, while those proteins were successfully recovered by anti-NGF treatment. The Gene Ontology (GO) and Kyoto Encyclopedia of Genes and Genomes (KEGG) enrichment analyses are conducted as well (Fig. 2c). Proteins in these two clusters are analyzed by using the STRING database, and the protein-protein interaction network is illustrated in Fig. 2d. Potential key proteins, in particular associated with cell-cell and PI3K-AKt signaling pathway, including proteins in cluster 3 (e.g., integrin beta-1 (ITGB1) and PIK3CA), as well as proteins in cluster 5 (e.g., laminin subunit alpha-4 (LAMA4), laminin subunit gamma-1 (LAMC1), integrin alpha-1 (ITGA1), Zyxin (ZYX), protein phosphatase 1 regulatory subunit-12B (PPP1R12B), and protein phosphatase 1 regulatory subunit-12C (PPP1R2C)), were detected. Creatine kinase M-type (CKM) was detected in cluster 3 as well.

### Expression Levels of ITGA1, ITGB1, LAMC1, and CKM in Uterus Were Detected by Western Blotting

**Fig. 3:**
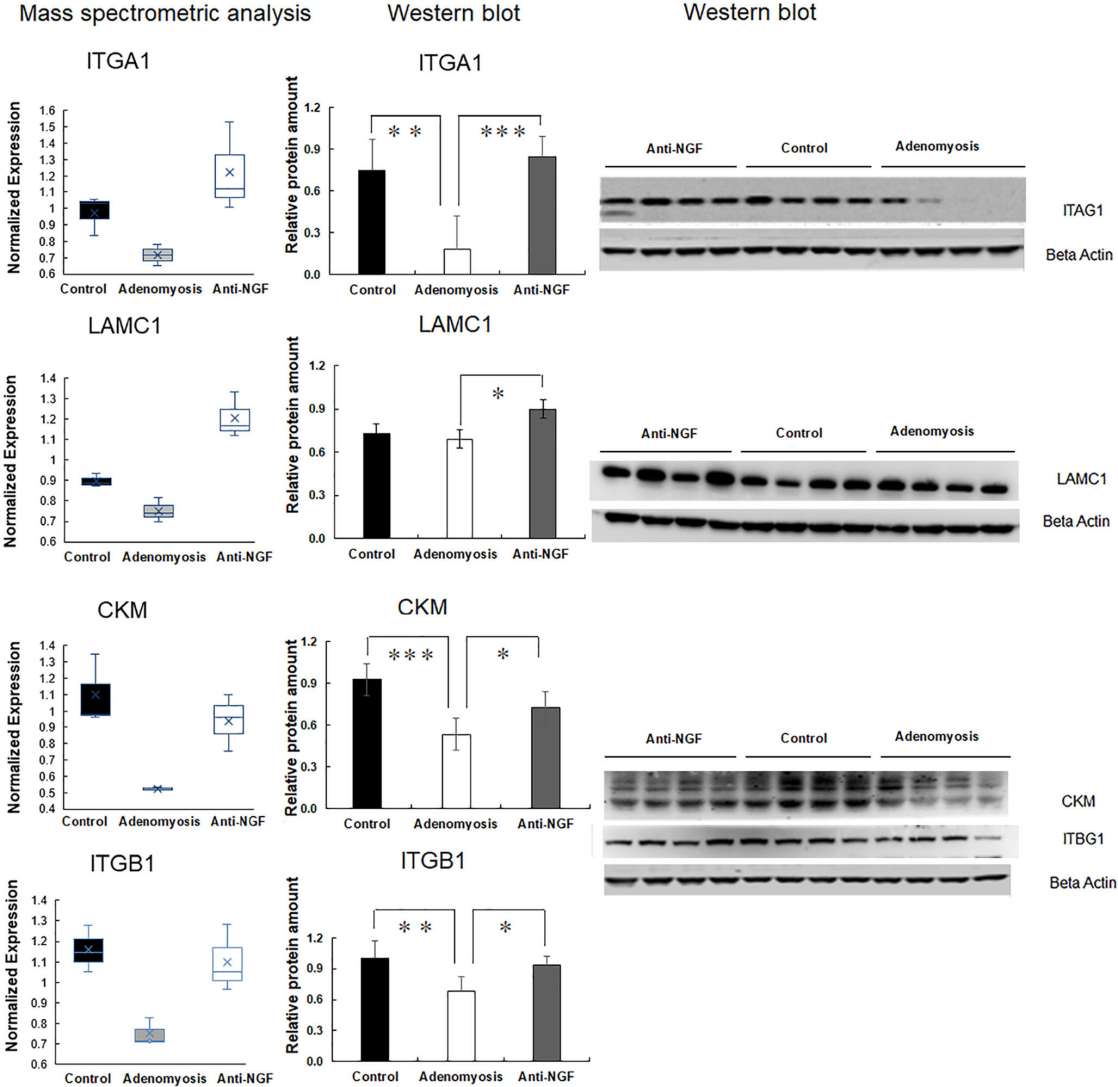
Western blot analysis of key proteins in adenomyosis group. Expression levels of ITGA1, ITGB1, LAMC1, and CKM were downregulated in the Adenomyosis group, while those were upregulated in anti-NGF group. *P < 0.05, **P < 0.01, ***P < 0.001

We conducted Western blotting to assay the expression levels of favorable proteins. The expression levels of ITGA1, ITGB1, LAMC1, and CKM are downregulated in the adenomyosis group compared with those in control group, while those proteins are successfully recovered by anti-NGF treatment (Fig. 3).

### Expression Levels of ITGA1, ITGB1, LAMC1, and CKM in Endometrial Tissue Were Detected by IHC

**Fig. 4:**
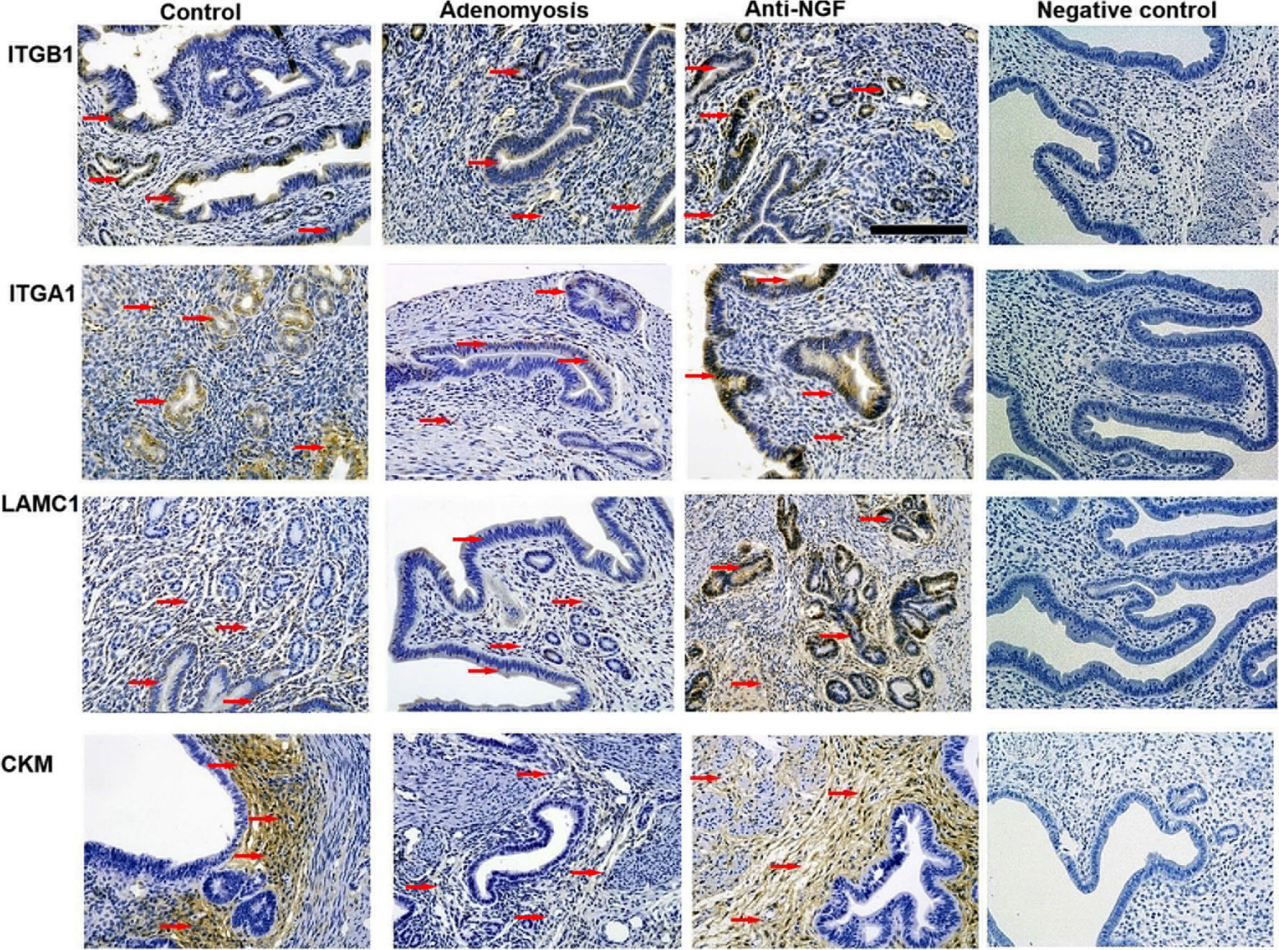
Immunohistochemistry (IHC) to detect the expression levels of ITGA1, ITGB1, LAMC1, and CKM in endometrial tissue of three groups. Immunoreactivity of ITGA1 and ITGB1 was predominantly investigated in the luminal epithelial cells and glandular cells, and they are also weakly expressed in stromal cells. Besides, immunoreactivity of LAMC1 was predominantly evaluated in the luminal epithelial cells, glandular cells, and stromal cells. The immunoreactivity of CKM was detected in endometrial stromal cells. (arrows, immunoreactive site; scale bar = 100 μm)

We further assessed the expression levels of ITGA1, ITGB1, LAMC1, and CKM in sections taken from the uterus. As illustrated in Fig. 4, immunoreactivity of ITGA1 and ITGB1 is predominantly investigated in the luminal epithelial cells and glandular cells and weakly investigated in stromal cells. Besides, immunoreactivity of LAMC1 was predominantly evaluated in the luminal epithelial cells, glandular cells, and stromal cells. The immunoreactivity of CKM was detected mainly in endometrial stromal cells. The four proteins exhibited a consistent trend with MS experiments, in which mice with experimentally induced adenomyosis had reduced expression level, while that was recovered after anti-NGF treatment.

## Discussion

In the present study, we established a mouse model of adenomyosis and explored the effects of anti-NGF therapy on the endometrial receptivity. Proteomic method was used to study therapeutic effects and mechanisms of several adhesion- and metabolic-related proteins. It was revealed that anti-NGF treatment slightly enhanced the embryo implantation rate in mice with adenomyosis. Furthermore, ITGB1, ITGA1, LAMC1, and CKM might participate in the recovery of adenomyosis.

Results of MS functional enrichment analyses showed that a group of proteins participated in cell-cell adhesion and metabolic processes, and changed in adenomyosis group, while were successfully recovered by anti-NGF treatment. Potential key proteins, associated with cell-cell adhesion and PI3K-AKt signaling pathway, are ITGB1, ITGA1, PIK3CA, LAMA4, LAMC1, ZYX, PPP1R12B, and PPP1R2C (Fig. 2c, d). For redox-related enzymes, the levels of aldehyde oxidase and superoxide dismutase were increased, while the levels of carbonyl reductase and alcohol dehydrogenase were decreased after anti-NGF therapy (see Supplementary Materials: MS analysis). In addition, the level of CKM was decreased in adenomyosis group whereas increased in anti-NGF group.

Integrin expression is characteristically increased in mid-luteal phase when implantation is expected to occur, serving as markers for the frame of the window of implantation [26]. The αvβ3 has been studied as a predictor of IVF success [27]. Integrin subunit α1 was observed on the glandular epithelium during the secretory phase of the menstrual cycle [28]. Besides, integrin subunits α2 and α3 were found to have a pericellular distribution, promoting cell-cell adhesion, as well as function of cells binding to components of the extracellular matrix (ECM), such as laminin and collagen [26, 28, 29]. Integrin subunit β1 was expressed in stromal cells around the implanted embryos on day 7, and it may strongly modulate decidualization of the endometrial stroma [30]. It was found that overexpression of integrin subunit β1 decreases the inhibition of miR-183 on the invasiveness of endometrial stromal cells, demonstrating that integrin subunit β1 is a significant factor (and a target of miR-183) for cell-cell adhesion and invasiveness [31]. In the present study, we found that integrin subunit α1 (ITGA1) and integrin subunit β1 (ITGB1) were decreased in adenomyosis group, while were elevated in Anti-NGF group, which might be beneficial for the embryo implantation.

LAMA4 and LAMC1 are the components of ECM. Sundqvist et al. found that the level of LAMC1 mRNA was decreased in the endometrium in patients with endometriosis compared with controls in the proliferative phase [32]. They thought that LAMC1 and other adhesion factors may play a role in the anchoring of endometrial cells at ectopic sites, thereby initiating the proliferation of these ectopic endometrial cells [32]. In the present study, the expression level of LAMC1 was not different between adenomyosis group and control group. Different from Sundqvist et al.’s findings, our samples were collected at postestrus phase. It was also prompt that LAMC1 might not play a vital role in impaired endometrial receptivity in mice with adenomyosis. However, Kaloglu et al. reported that fibronectin and laminin have dynamic expressions related to the morphological differentiation of endometrial stroma, prompting their role in regulation of trophoblast invasion and adhesion [33]. Thus, it can be concluded that anti-NGF therapy, improving the expression level of LAMC1, may possess advantages for embryo implantation.

Additionally, PI3K/AKT signaling pathway plays a pivotal role in keeping balance between cell survival and apoptosis [34]. PIK3CA was found participated in the process of gland development and epithelial cell invasiveness, which are of great importance in the pathology of adenomyosis [35] [36]. PPP1R12C and PPP1R12B are the main members of myosin-targeting subunit (MYPT), which is a major component of the phosphatase and a crucial determinant of function of smooth muscle cells [37]. In the present study, the low expression levels of PPP1R12C and PPP1R12B were partly related to the myometrium injury in adenomyosis, and anti-NGF therapy might be advantageous for repair of myometrial defect. However, to date, a limited number of studies have reported the relationship between MYPT and endometrial receptivity.

In the current study, we found that the levels of aldehyde oxidase and superoxide dismutase were increased after anti-NGF therapy. Superoxide dismutase (SOD) is an enzyme that alternately catalyzes the dismutation (or partitioning) of the superoxide (O_2_^−^) radical into either ordinary molecular oxygen (O_2_) or hydrogen peroxide (H_2_O_2_). The elevation of the expression level of SOD indicated that anti-NGF treatment may alleviate oxidative stress in uteri. In addition, the level of CKM was decreased in adenomyosis group, while that was increased in anti-NGF group. The traditional understanding of biological function of CKM is on the basis of energy reserve and ATP synthesis [38]. Scholars demonstrated that through CrP/CK, the creatine shuttle plays a vital role in local delivery of ATP during the preparation for cytokinesis in the preimplantation embryos [39]. Additionally, CKM may reflect a condition of inflammation or oxidative stress. A previous research reported that inflammatory parameters were associated with decrease of CK [40]. In another study on skeletal muscle, oxidative modification was found to decrease the activity of CKM [41]. In the current study, we noted a crucial role of CKM during onset and recovery of adenomyosis, which indicated that anti-NGF treatment could improve the energy metabolism of uterine adenomyosis in mice. We speculated that anti-NGF treatment partly increased the expression level of CKM due to its influences on reducing inflammation and anti-oxidation.

There are few studies on the influence of NGF or other neurotrophic factors on endometrial receptivity. Based on the present study, it is speculated that patients with increased local secretion of NGF, such as adenomyosis [14] or endometriosis [42], anti-NGF therapy may improve their pregnancy outcome slightly. The main clinical effect of NGF antibody was pain inhibiting by reducing the production of inflammatory factors [43]. As it known that, the dysregulation of the inflammation pathway participates in endometriosis- or adenomyosis-associated infertility, which alters endometrium receptivity by imparing biochemial responses (e.g., embryo attachment, decidua invasion) and decidualization [44, 45]. The effect on alleviating inflammation by anti-NGF influences positively the adhesion and metabolism factors. We conducted Western blotting and IHC to assess the levels of favorable proteins (ITGB1, ITGA1, LAMC1, and CKM). These proteins were participated in adhesion and metabolism as well as were recognized involved in the endometrial receptivity in previous studies.

However, the present study contains limitations. Firstly, the pregnancy rate was generally low, which might be partly due to aging. We selected the period of 29–30 weeks due to the characteristics of the established mouse model of adenomyosis [16]. The immunological reaction due to different mice stains from donors to surrogates might reduce implantation/pregnancy, too. The low pregnancy rate and insufficient samples size reduced the differences among three groups. Secondly, we used uterine tissues for MS analysis. The myometrium of adenomyosis mice was thin and disordered, making it difficult to distinguish endometrium from myometrium. Eventually, further clinical research needs to be conducted to indicate whether anti-NGF has the potential embryotoxicity.

As a new pain relief medicine, NGF-neutralizing antibody is highly promising for relieving chronic pain. Two anti-NGF antibodies, tanuzemab and fasinumab, are in active development, with tanuzemab close to completing phase 3 trials in preparation for an application for approval for clinical use [43]. Our findings suggested that intervention of adenomyosis-related inflammatory response by the NGF-neutralizing antibody is beneficial to improve endometrial receptivity. It means that pharmaceutical interventions targeting NGF may provide a possible novel therapeutic strategy for treating adenomyosis-related infertility.

## Conclusions

Anti-NGF therapy was proved to improve fertility of mice with experimentally induced adenomyosis, possibly through promoting integrin-related proteins. However, further clinical research needs to be conducted to indicate whether anti-NGF has the potential embryotoxicity.

## Electronic supplementary material

Supplementary Materials: Proteomic analysis.xlsx.

ESM 1 (XLS 3799 kb)
